# The establishment of a regression model from four modes of ultrasound to predict the activity of Crohn's disease

**DOI:** 10.1038/s41598-020-79944-1

**Published:** 2021-01-08

**Authors:** Jigang Jing, Yuting Wu, Hu Zhang, Yan Zhang, Jingxi Mu, Yan Luo, Hua Zhuang

**Affiliations:** 1 Department of Ultrasonography, West China Hospital of Sichuan University, Chengdu, China; 2 Department of Gastroenterology, West China Hospital of Sichuan University, Chengdu, China; 3 Center for Inflammatory Bowel Disease, West China Hospital, Sichuan University, Chengdu, China

**Keywords:** Gastrointestinal diseases, Ultrasonography

## Abstract

To establish a multi-parametric regression model from four modes of ultrasound to predict the activity of Crohn's disease (CD) noninvasively. Score of 150 of the Crohn’s Disease Activity Index (CDAI) was taken as the cut-off value to divide the involved bowel segments of 51 patients into the active and inactive group. Eleven parameters from four modes of ultrasound (B-mode ultrasonography, color Doppler flow imaging, contrast-enhanced ultrasonography and shear wave elastography) were compared between the two groups to investigate the relationship between multimodal ultrasonic features and CD activity. *P* < 0.05 was considered statistically significant. Parameters with AUC larger than 0.5 was selected to establish the prediction model of CDAI. Totally seven ultrasound parameters (bowel wall thickness, mesenteric fat thickness, peristalsis, texture of enhancement, Limberg grade, bowel wall perforation and bowel wall stratification) were significantly different between active and inactive group. A regression model was established based on the seven parameters as followed: CDAI = 211.325 + 3.186BWT − 53.003BWS + 6.280BWP + 0.392MFT + 22.239PS + 79.012LG + 72.793TE. (R^2^ = 0.72, *P* = 0.037). The multimodal ultrasound parametric regression model was designed to predict CDAI score invasively. The model has the potential to provide an alternative method for quantifying the CD activity.

## Introduction

Crohn’s disease (CD) is one of inflammatory bowel diseases (IBD), which is a chronic inflammatory state with recurrent attack and remission caused by chronic gastrointestinal mucosal immune system disorders^1^. Its incidence is relatively high in European and American countries, while it in China increases steadily year by year^2^.

Assessment of CD activity has important clinical value in reflecting its severity, estimating prognosis and outcome, selecting therapeutic schedule, and evaluating the efficacy^3^. Due to the particularity of pathology and long course of CD, the active and inactive phase alternately repeat, the interval varies from person to person, so the disease activity needs to be monitored dynamically.

The main means of evaluating CD activity include clinical activity score, laboratory examination, colonoscopy and imaging examination. Crohn’s Disease Activity Index (CDAI) is the most commonly used tool in clinical practice^4^. About laboratory examination items, C-Reactive Protein (CRP) and erythrocyte sedimentation rate (ESR) cannot reflect the local condition of the involved bowel. CRP is less useful as a disease activity marker in patients with ileal CD than those with ileocolonic or colonic CD, which was reported to be normal in active ileal CD cases^5^. Colonoscopy can accurately evaluate the mucous membrane on the inner surface of the bowel, discover superficial ulcerations and scar healing, and obtain pathological diagnosis, but can’t evaluate the transmural abnormalities^6^. In the case of stricture or bowel adhesion, colonoscopy cannot reach the proximal end of the stenosis, thus the bowel segment can’t be assessed.Cross-sectional imaging techniques are playing an increasing role in the evaluation of suspected small bowel disorders. Computed tomography enterography (CTE) can reveal the pathological changes of luminal, mural and extramural features, but CD with long duration and recurrence requires close follow-up, and CTE is not suitable for routine surveillance due to its radiation. Magnetic resonance enterography (MRE) can obtain similar information as CTE. MRE is more time-consuming, needs more patient compliance and its image quality varies to a greater extent than CTE, which restricts the wide application of MRE^7^. Some reports believed that ultrasound was a reliable and non-invasive method for early diagnosis of CD^4,8,9^. Bowel ultrasound has already been routinely used in following up of CD. In 2018, the European Federation of Societies for Ultrasound in Medicine and Biology (EFSUMB) recommended B-US for evaluation of CD activity^10^. In 2019, European Crohn’s and Colitis and the European society of gastrointestinal and abdominal radiology (ECCO-ESGAR) released the guide for diagnosis and assessment of IBD^11^. In the guide, ultrasound was reported to be one of the reliable cross-sectional imaging techniques for assessment of CD activity.

Ultrasound is convenient, non-invasive and radiation-free, which has become a common method to evaluate the condition of CD since 1979^12^. To our knowledge, there are few reports about how to use multi-parameters of all ultrasonic modes, including B-mode ultrasonography (B-US), color Doppler flow imaging (CDFI), contrast-enhanced ultrasonography (CEUS) and shear wave elastography (SWE) to evaluate CD activity systematically. With the widespread adoption of CEUS and SWE, it is worth further exploration to use multimodal multi-parameter ultrasound to assess CD activity.

We made a pilot study about the question that multimodal ultrasound can or not reflect the change of CD activity timely, which is the fundamental that ultrasound has or not the potential to provide an alternative method for quantifying the disease severity after treatments.

## Materials and methods

### Clinical data collection

The patients with clinical diagnosis of CD by the criteria for Crohn's disease recommended by the World Health Organization since 1975 (updated in 2015) in West China Hospital of Sichuan University from March 2018 to January 2019 were prospectively collected. Data of Clinical information, ultrasound, colonoscopy and pathological examination was collected. Prior to the start of the study, the ethics review was passed at West China Hospital of Sichuan University (No. 339, 2018). Informed consent was obtained from all participants and/or their legal guardians. All experiments were performed in accordance with relevant named guidelines and regulations. The inclusion criteria were CD had been confirmed by the department of gastroenterology, West China Hospital of Sichuan University. The exclusion criteria were as follows: 1) Patients with other intestinal inflammation and tumors confirmed by colonoscopy, clinical findings or pathological biopsy; 2) patients with incomplete ultrasound data (including missing data of B-US, CDFI, CEUS and SWE). All patients were graded according to the best-CDAI scoring standard developed by America National Cooperative Crohn's Disease Study group^13^. A score of 150 of the Crohn’s Disease Activity Index (CDAI) was used as the cut-off value of activity and inactivity, and the involved bowel segments were divided into active group (CDAI ≥ 150) and inactive group (CDAI < 150). The time interval from the point that we made a CDAI assessment to the corresponding point that we performed the ultrasound examination was less than 1 week.

### Ultrasound assessment

**Figure 1:**
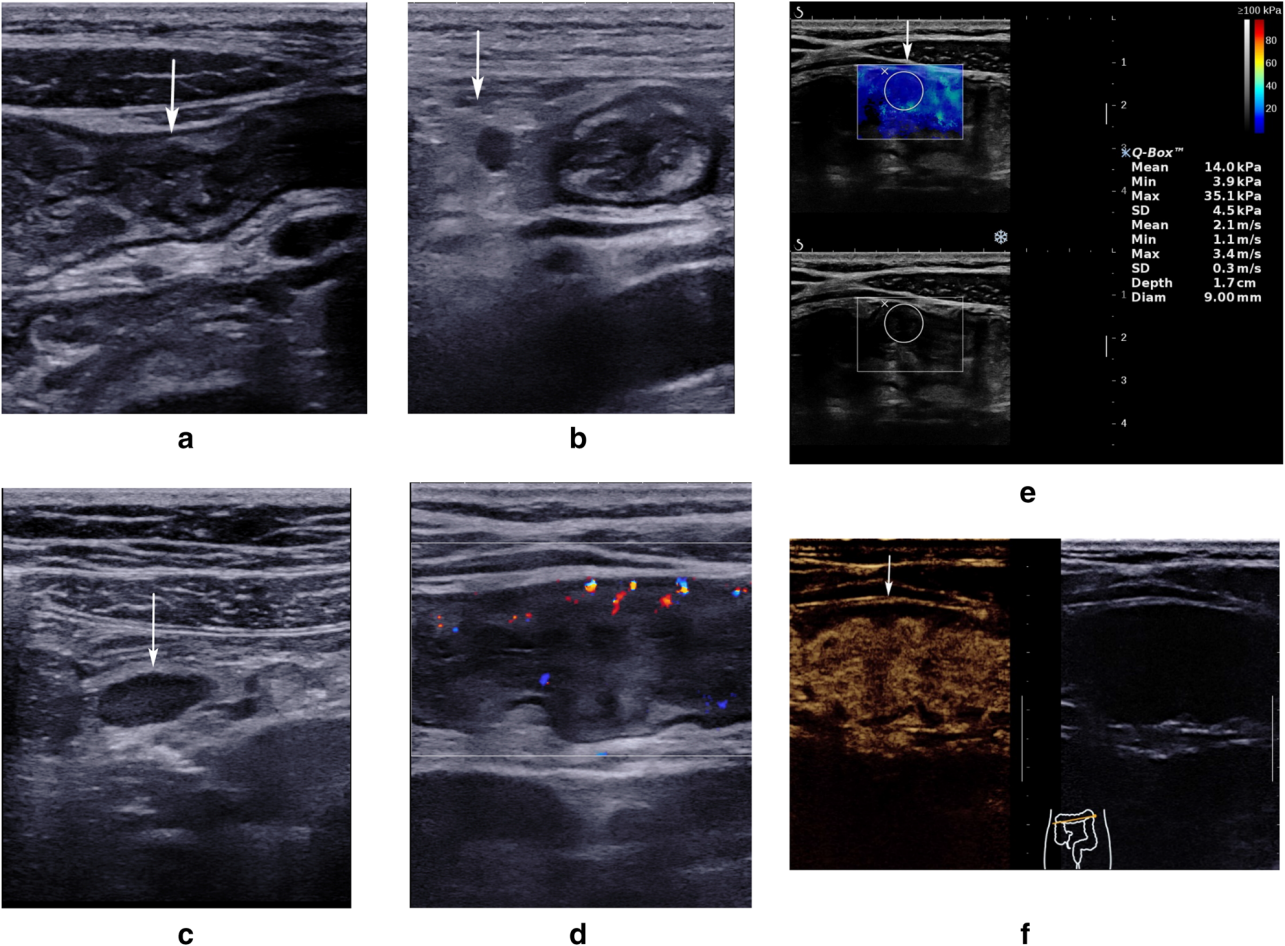
Examples of ultrasonic findings. Male, 18y, recurrent gastrointestinal bleeding for 1 year, CDAI: 336. (**a**) White arrow indicates thickening of the colon wall. (**b**) White arrows indicate mesenteric fat thickening. A lymph node can be detected in the thickened mesenteric fat. (**c**) White arrow indicates mesenteric lymphadenopathy. (**d**) The vascularization in the colon wall was scored to be Limberg II. (**e**) White arrow indicates that the anterior wall of the colon is soft, and the shear wave elastography of the anterior wall is homogeneous blue with a mean SWV of 2.1 m/s. Figure 1f. White arrow indicates a transmural homogeneous enhancement (TE homogeneous), and stratification of the intestinal wall still remains (SE presence).

The Aixplorer US system (SuperSonic Imagine, France) equipped with shear wave elastography (SWE) was used. The patients were examined after the ingestion of 1000 mL of iso-osmolar polyethylene glycol (PEG) with a convex transducer (C6-1) and line convex transducers (L10-2, L15-4). (Examples of ultrasonic findings see Fig. 1).

B-US findings were recorded as a series parameters including number and location of lesions, the maximum bowel wall thickness (BWT, BWT > 3 mm was considered to be thickened) of the affected segments (Fig. 1a), bowel wall stratification (BWS), bowel wall penetration (BWP), peristalsis (PS), minimal luminal diameter (D, D < 10 mm was considered to be with a stricture) , mesenteric fat thickness (MFT) (Fig. 1b), lymph nodes (LN) (LN enlargement was defined as the maximum diameter greater than 5 mm) (Fig. 1c). All ultrasound parameters were recorded for each segment of the lesions.

CDFI was performed to observe the bowel wall vascularization and grade them according to the Limberg scoring system (LG)^8^. Results are recorded into 5 grades (LG 0, I, II, III and IV), LG 0, I, II was defined as a lower level of blood flow (Fig. 1d), while LG III and IV was defined as a higher level of blood flow.

SWE was performed. An average of shear wave velocity (SWV) of five points was obtained, which were uniformly distributed in the thickened bowel wall (Fig. 1e).

After the rapid injection of 2.4 mL contrast agent (Sono*Vue*, Bracco, Milan, Italy), the process of CEUS was observed, including stratification of enhancement (SE) and texture of enhancement (TE) (Fig. 1f). The results of SE were recorded as presence or absence. And those of TE were recorded as homogeneous or heterogeneous.

All cases were scanned thoroughly by one doctor (J.JG.). A total of 11 ultrasound parameters for each bowel segment was obtained, including BWT, BWS, BWP, D, MFT, PS, LN, LG, SWV, SE and TE. Of the 11 parameters, values of continuous variables (such as BWT, D and MFT) were recorded, positive or negative results of binary variables (such as BWP, BWS and PS) were recorded as 0 or 1.

### Single factor and multi-factor analysis of the relationship between ultrasound parameters and CD activity

SPSS software (version 24.0, SPSS Inc., Chicago, USA) was used for statistical data processing and analysis. Enumeration data were represented by frequency and rate, and measurement data were represented by Mean ± SD. For single ultrasound parameter of CD multimode imaging, t test and *x*^2^ test were used for inter-group comparison according to different data types. *P* < 0.05 was considered statistically significant, and all were bilateral tests. Eleven ultrasound parameters in CD lesions in active group and inactive group were compared. The parameters significantly different between the two groups (with a *P* value less than 0.05) were selected to draw receiver operating characteristic (ROC) curves. Parameters with areas under the curve (AUC) greater than 0.5 were included to establish the multiparametric regression prediction model.

## Results

### Clinical manifestations and main ultrasound findings

A total of 79 involved bowel segments were included, coming from 51 patients. Demographic and clinical data and findings of other medical imaging (CTE/MRE) were shown in Table 1. Colonoscopy and histopathological data were shown in Table 2.

**Table 1:** Demographic and clinical data of 51 cases.

Characteristics	Data
Gender (male/female)	29/22
Age (years)	34.68 ± 10.68 Range:18–62
Course of disease (months)	45.26 ± 60.64 Range:0.5–360
CDAI	240.73 ± 73.88
**Laboratory examination**	
CRP (mg/L)	31.87 ± 39.86
ESR (mm/h)	38.37 ± 28.88
**Symptom (n, %)**	
Abdominal pain	28 (54.9%)
Diarrhea	17 (33.3%)
Bloody stools	9 (17.6%)
Shapeless stool	4 (7.8%)
Fever	4 (7.8%)
Intestinal obstruction	6 (11.8%)
perianal abscess and anal fistula	8 (15.7%)
Epigastric discomfort	1 (2.0%)
Constipation	1 (2.0%)
**Findings of CT/MRI (n, %)**	
Thickening and enhancement of the bowel wall	33 (64.7%)
Perforations, fistulas and sinus	5 (9.8%)
Stricture	8 (15.7%)
“Comb pattern” of mesenteric angiogenesis	2 (3.9%)
Iliopsoas abscess	1 (2%)
Obstruction	2 (3.9%)
**Therapeutic method (n, %)**	
Mesalazine	6 (23.6%)
Mesalazine after antituberculosis therapy	6 (11.8%)
Hormones and immunosuppressants	17 (33.3%)
Infliximab	9 (17.6%)
Surgical treatment	13 (25.5%)

**Table 2:** Laboratory examination, colonoscopy and histopathological data of 51 cases.

Examination	Data
**Colonoscopy**	
Ulceration	24 (47.2%)
Mucosal nodule	6 (11.8%)
Scar	9 (17.6%)
Other findings	12 (23.5%)
**Histopathological**	
Moderate or severe inflammation	19 ( 37.2%)
Mild inflammation	17 (33.3%)
Chronic inflammation	13 (25.5%)
Other findings	4 (7.8%)

Ultrasound findings of 51 CD patients with different activity status were shown in Table 3.

**Table 3:** Main US findings of four modes of CD lesions with different activity.

Parameters	Active group (n = 71)	Inactive group (n = 8)	*P*
**B-US**			
BWS			< 0.05
PR	58	7	
AB	13	1	
BWP			< 0.05
PR	3	0	
AB	68	8	
PS			< 0.05
PR	24	3	
AB	47	5	
LN			> 0.05
Enlarged	31	3	
Normal	40	5	
BWT (mm)	8.04 ± 3.32	6.75 ± 2.51	< 0.05
D (mm)	1.31 ± 2.16	2.75 ± 3.24	> 0.05
MFT (mm)	10.49 ± 5.09	8.44 ± 5.21	< 0.05
**CDFI**			
LG			< 0.05
LG 0, I, II	50	7	
LG III, IV	21	1	
**SWE**			
SWV (m/s)	2.16 ± 0.41	2.36 ± 0.44	> 0.05
**CEUS**			
SE			> 0.05
PR	58	5	
AB	13	3	
TE			< 0.05
HET	8	0	
HOM	53	8	

(1) n is the number of the involved bowel segments; (2) divided into active group (CDAI ≥ 150) and inactive group (CDAI < 150); (3) Meanings of abbreviations: BWT (Bowel wall thickness), BWS (Bowel wall stratification), BWP (Bowel wall perforation), D (intestinal lumen diameter), MFT (Mesenteric fat thickness), PS (bowel peristalsis), LN (lymph nodes enlargements), LG (Limberg grade of the bowel wall vascularization), SWV (shear wave velocity), SE (presence of the stratified enhancement of the bowel), TE (existence of the homogeneous enhancement of the bowel wall), HET (heterogeneous), HOM (homogeneous), PR (presence),AB (absence).

### Relationship between Ultrasound parameters and CDAI

As to the relationship between multimodal multiparametric ultrasound and CDAI, 11 ultrasound parameters were extracted for analysis. Seven (7/11) ultrasound parameters (BWT, BWS, BWP, PS, MFT, LG, TE) were significantly different between active and inactive group (*P* < 0.05). ROC curves of the 7 significantly parameters were drawn, which were also with AUC larger than 0.5 (Table 4). While others (LN, SWV, SE, D) were not significantly different, which means they were not correlated with Crohn' s disease activity (*P* > 0.05).

**Table 4:** Area under ROC curve for 7 ultrasound parameters.

Ultrasound parameters	BWT	BWS	BWP	PS	LG	TE	MFT
AUC	0.636	0.529	0.521	0.511	0.558	0.556	0.622

Meanings of abbreviations: BWT (Bowel wall thickness), BWS (Bowel wall stratification), BWP (Bowel wall perforation), PS (bowel peristalsis), LG (Limberg grade of the bowel wall vascularization), TE (existence of the homogeneous enhancement of the bowel wall), MFT (Mesenteric fat thickness).

### Establishment of the regression model to predict CDAI

The regression model of CDAI was established based on the 7 parameters (R^2^ = 0.72, F = 2.204, *P* = 0.037):

CDAI = 211.325 + 3.186BWT − 53.003BWS + 6.280BWP + 0.392MFT + 22.239PS + 79.012LG + 72.793TE.

The absolute values of the regression coefficient of 7 parameters in an ascending order were as followed: LG, TE, BWS, PS, BWP, BWT and MFT. Therefore, LG, TE, BWS and PS had more impact on the regression model.

## Discussions

B-US, CDFI and CEUS are the main mode of ultrasound to evaluate the activity of CD^14–17^. SWE is firstly used to judge bowel wall fibrosis, but not the status of activity of CD^18–21^. In 2017, Novak et al*.* reported a simplified scoring method to assess the activity of CD combining B-US and CDFI together^22^, which was not widely accept by the clinic. In fact, there are few studies combining all Ultrasound techniques together, including B-US, CDFI, CEUS and SWE, focusing on the ultrasonic scoring system to assess the activity of CD.

In this study, totally 11 ultrasound parameters out of four modes (B-US, CDFI, CEUS and SWE) were obtained. At last, a regression model based on 7 significant parameters was established to reflect the activity status (active or inactive). And the assessment by our model is non-invasive and quantitative.

In 1979, some scholars focused on the relationship between BWT and CD^23^. The bowel wall is thickened due to swollen loose connective tissue and dilatation of vessels and lymphatics when inflammation occurs^24^. In some studies, BWT > 3 mm or BWT > 4 mm were used as the diagnostic criteria for CD, and their sensitivity versus specificity were 88% ~ 75% versus 93% ~ 97%, respectively^25^. We used BWT > 3 mm as the cut-off value in this study, and the average BWT is 7.9 mm. Previous studies showed inconsistent results on BWT cut-off value to differentiate active and inactive CD^22,26^. Literatures reported that the average BWT ranged from 5.1 ± 1.5 to 6.47 ± 2.03 mm in the active CD group and from 3.3 ± 1.6 to 5.12 ± 1.68 mm in the inactive group. The data of our study showed that the difference of BWT between active and inactive group was statistically significant. In this study, the median BWT in active and inactive group was 8.04 ± 3.32 mm and 6.75 ± 2.51 mm, respectively, larger than that was previously reported^27–29^. This could be associated with a longer course of the disease in our study (average course of disease was about 45 months), and pathological examination showed that severe inflammation accounted for 37.2% in our cases.

The results showed that BWP in the active group and inactive group was statistically significantly different. Accurate diagnosis of bowel wall perforation provides strong support for active CD. Previous reports believed that the ultrasound diagnosis accuracy of bowel wall perforation was 50% ~ 95.7%^30–32^. Fistulas are the result of bowel wall perforation, which are the hypoechoic structures between intestines, intestines and internal organs or intestines and skins, containing liquid or gas. Both Gasche et al*.* and Pallotta et al*.* agreed that hypoechoic peri-intestinal lesions with diameter below 20 mm were regarded as fistulas^31,32^. Maconi et al*.* reported that MFT and CDAI were correlated significantly^33^, and the presence of lymph node enlargement was weakly correlated with the clinical and biochemical activity of CD^34^. Our results showed that the MFT of the active and inactive group was statistically significantly different. There was no statistically significant difference in lymph node enlargements between the active and inactive group, which was consistent with that of Maconi et al. The reason might be that lymph node enlargement is mostly caused by reactive hyperplasia, not only due to inflammation.

The Limberg scoring system was established to evaluate intestinal bowel vascularization by Limberg B since 1999^8^. Our data showed that between the active and inactive group, there was a statistically significant difference of LG. The higher Limberg grade occurred more in active group (active group/inactive group: 21/1) than in inactive group. That is to say, the status of CD activity can be determined according to the bowel blow flow pattern.

SWE imaging is an emerging diagnostic quantitative technique, which was widely used in different diseases. In theory, compared with the normal bowel wall, the involved wall was relatively hard, especially the one with obvious fibrosis. In our study, SWE examination was performed in all involved bowel segments, and the mean SWV value was 2.22 m/s. Dillman et al*.*^19^ reported a higher SWV (1.87 ± 0.44 m/s) in high-grade fibrosis lesions than in low-grade fibrosis lesions (1.50 ± 0.26 m/s). Furthermore, the reason for our results with the high measurements of elastography was likely related to the hardening of bowel wall fibrosis caused by a longer course (the average course of CD in our study was 45.3 months). Fufezan et al*.*^35^ evaluated the activity of pediatric CD with elastography and proposed a scoring system on the basis of the classification of three specific bowel wall patterns. Their result showed that there was statistically significant correlations between the bowel wall changes, presence of complications, activity markers and the elastography score. Our results indicated that there was no statistically significant difference in SWV between the active group and the inactive group, suggesting that there was no significant correlation between CDAI and SWV. This was consistent with the results reported by Goertz et al*.*^36^ that there was no correlation between ARFI SWV and clinical activity. The possible reason is that CDAI based on the clinical symptoms may not absolutely reflect the pathology of bowel wall.

In patients with active CD, the microvascular density of the bowel wall may increase due to the proliferation of micro-vessels in inflammation process. CEUS for evaluation of inflammatory bowel disease is widely accepted and recommended in the guidelines updated by EFSUMB^37^. CEUS can detect the blood flow in the involved bowel wall more sensitively than CDFI. It has been reported in the literature that there was a good direct correlation between the peak intensity of CEUS and bowel wall thickness^15^. But it is also influenced by the dose of contrast agents and patients’ cardiac function. So previous studies failed to reach a consistent conclusion^38,39^. In our study, we selected two parameters of CEUS (SE and TE) to analyze. Presence of SE were revealed by CEUS in most involved bowel segments with or without an active inflammatory state. The results showed that there was no statistically significant difference in SE between the active group and the inactive group. That is to say, SE can’t tell CD is active or not. However, the difference in TE was statistically significant, which may suggest that the texture of enhancement of inflammatory bowel wall can reflect the state of lesion activity.

Pascu et al*.*^38^ pointed out that intestinal ultrasound findings (wall thickness, wall stratification, Doppler signal, compressibility and peritoneal surface thickening) were related to activity, but they didn’t analyze and confirm the impact of different ultrasound parameters. In this study, the regression coefficients of MFT and BWT were 0.39 and 3.186, respectively. The results of this study suggest a correlation between MFT, BWT and activity. That is consistent with Maconi et al*.*’s results^34^. It was found that in the ultrasound parameters regression model with CDAI taken as the reflector of CD activity, the parameters with high regression coefficients were LG, TE, BWS and PS (79.012, 72.793, -53.003 and 22.239). That is to say, LG, BWS, PS and TE have more impact on predicting CD activity. They should be the key factors.

We recognized our limitations were lack of surgical results in cases, and that the reference indicator of CD activity in this study was CDAI, which is the reflection of the systemic inflammation rather than local involvements of bowel segments. Our study was a single-center study and we planned to expand the sample size with other units and verify our regression model.

In our study, we proposed a regression model based on multimodal multi-parametric ultrasound to assess CDAI. The predictive value (R^2^) is 0.72. This shows that the multi-parametric regression model based on multimodal ultrasound can predict the activity of CD to some extent. But its value needs to be further verified by increasing cases.
